# Altered neural oscillatory dynamics underlie reduced anticipatory schema use during event segmentation in adolescents with High-Functioning Autism Spectrum disorder

**DOI:** 10.1016/j.nicl.2026.103998

**Published:** 2026-04-26

**Authors:** Ronja Limburg, Michel Benjamin Kopp, Xianzhen Zhou, Foroogh Ghorbani, Veit Roessner, Bernhard Hommel, Christian Beste, Astrid Prochnow

**Affiliations:** a Cognitive Neurophysiology, Department of Child and Adolescent Psychiatry, Faculty of Medicine, TU Dresden, Schubertstrasse 42, 01307 Dresden, Germany; b German Center for Child and Adolescent Health (DZKJ), partner site Leipzig/Dresden, Dresden, Germany; c School of Psychology, Shandong Normal University, No. 88 East Wenhua Road, Jinan 250014, Shandong Province, China

**Keywords:** Adolescent, Beamforming, Electroencephalography, Prediction error, Working memory, Event perception

## Abstract

Autism Spectrum Disorder (ASD) is characterized by diverse symptoms that may stem from atypical integration of sensory information and prediction processes. Event Segmentation Theory (EST) offers a framework to examine how individuals parse continuous experience into meaningful units. This study investigated whether altered event segmentation in adolescents with ASD arises from atypical neural oscillatory dynamics underlying prediction and updating processes.

Behaviorally, adolescents with ASD showed a weaker adaption of segmentation behavior to the occurrence of situational changes than neurotypical participants, particularly in response to small spatial changes. Neurophysiologically, both groups exhibited alpha and beta power reductions at event boundaries, but these modulations were less extensive and more localized in ASD. Source-level theta modulations before boundaries were only present in ASD, suggesting increased reliance on error monitoring rather than event schema access.

Adolescents with ASD show reduced event segmentation in response to contextual changes accompanied by altered alpha, beta and theta oscillatory dynamics, indicative of reduced schema-based integration, inflexible updating of event representations and heightened conflict monitoring. These findings highlight event segmentation as a potential cognitive mechanism contributing to broader ASD symptomatology.

## Highlights

• Explores Event Segmentation Theory as a framework to explain deficits in ASD.
• Uses a naturalistic event segmentation paradigm.
• Identification of distinct oscillatory dynamics before and after event boundaries.
• Reduced adaption of segmentation behavior to situational changes in ASD.
• Indication for an atypical balance of error monitoring and event-schema use in ASD.

## 1 Introduction

Autism Spectrum Disorder (ASD) is a complex neurodevelopmental disorder, resulting in lifelong impairments for some patients (van Heijst and Geurts, 2015). While research on ASD often centers on specific symptoms (Chmielewski and Beste, 2015, Sharma et al., 2018), knowledge on the underlying cognitive mechanisms is limited. In the current study, we tested whether Event Segmentation Theory (EST) (Zacks, 2020, Zacks and Swallow, 2007) may provide a novel and suitable framework to capture and explain deficits seen in ASD.

EST proposes a model of how people parse the continuous and dynamic inflow of information into discrete meaningful units, called event segments (Kurby and Zacks, 2008, Zacks et al., 2007, Zacks and Sargent, 2010). This segmentation relies on *working event models* – working memory representations of ongoing situations – which are shaped by sensory input and expectations informed by *event schemata*. Event schemata comprise long-term memory contents about the to-be-expected unfolding of events based on prior experience with comparable situations (Zacks et al., 2007). Predictions about the near future are based on the event models and are continuously compared to sensory information of what is actually happening. Potentially resulting prediction errors are integrated over time, and if the error signal becomes too high (indicating an inadequacy of the event model for the current situation), the event model is updated, and an event boundary is set (Kurby and Zacks, 2008, Zacks et al., 2007, Zacks and Sargent, 2010). After an event boundary, a new working event model has to be established based on environmental information and appropriate event schemata (Kurby and Zacks, 2008, Zacks et al., 2007, Zacks and Sargent, 2010, Zhou et al., 2024).

To provide a practical example that makes the event segmentation process more tangible, consider a typical day at work. After arriving at the office, you might begin checking your During this activity, your perceptual input (a computer screen and keyboard), intention (responding to messages), and action patterns (reading, typing) remain relatively consistent and allow you to anticipate the unfolding of the near future (e.g., opening the next e-mail, reading it, and replying to it). In this situation, a stable working event model can be maintained. In contrast, an event boundary would be triggered whenever a substantial shift in one or more dimensions defining the situational context occurs, such as locations, social context, intentions or action structure (Zacks et al., 2009). For example, a colleague might approach you with a question (change in social context), you might decide to work in a library because there is a loud construction site in front of your window (change in location), or you might have responded to all e-mails and commence the next task (change in intention and action pattern). These changes generate prediction errors and signal the need to update the working event model.

Notably, processes described in EST are tightly linked to cognitive functions (Zacks et al., 2007, Zacks and Swallow, 2007) that show apparent divergences in ASD, including attention (Chien et al., 2014), working memory (Habib et al., 2019, Wolff et al., 2018), episodic memory (Geng et al., 2024) and cognitive control (Chmielewski et al., 2016, Chmielewski and Beste, 2015, Solomon et al., 2008). Based on reports of idiosyncratic perception and processing in ASD, a distinct manifestation of altered event segmentation can be expected. Predictive coding accounts characterize ASD by an exaggerated sensitivity to input variability, with expectations based on context, such as event schemata, being insufficiently considered (Goris et al., 2018, Schreiter and Beste, 2020, Van de Cruys et al., 2017). Similarly, a common feature in ASD is a heightened focus on details, often at the expense of the ability to contextualize information. Consequently, the capacity to generalize and recognize similarities is compromised (Happé and Frith, 2006, Plaisted, 2001). This is also highlighted in accounts linking ASD symptoms to overly rigid prediction ability (Chmielewski and Beste, 2015, Gomot and Wicker, 2012, Plaisted, 2001, Schreiter and Beste, 2020, Sinha et al., 2014). Such impaired prediction may explain both context-inappropriate responses and the strict adherence to routines in ASD, as routines ease the anticipation of upcoming events and foster a sense of control (Gomot and Wicker, 2012, Sinha et al., 2014). However, even small deviations can disrupt this sense of control leading to the commonly reported distress of ASD patients when confronted with change (Berenbaum et al., 2008, Boulter et al., 2014, Sinha et al., 2014). In terms of event segmentation, the disproportional weighting of prediction errors and detail-focused perception without recognizing global concepts might ultimately result in more frequent updating of the current event model. Given difficulties in interpreting nonverbal communicative signals and the overall reduced social interest in ASD (American Psychiatric Association & American Psychiatric Association, 2013), atypical segmentation could also result from a particular impairment in recognizing meaningful shifts in the social dynamic of events (von Hofsten et al., 2009). Importantly, alterations of event segmentation in ASD might not only occur when it comes to detecting the end of an event segment, but these cognitive functions are also involved in establishing a new event segment after an event boundary. We hypothesize that altered event segmentation in ASD compared to neurotypical (NT) peers is observable in three ways: (I) individuals with ASD segment more frequently in response to minor changes in situational details, while (II) being less responsive to changes affecting a situation’s overall meaning. (III) The latter tendency should be particularly pronounced when such contextual changes can only be detected by considering social aspects and their significance in determining the broader situational context.

To move beyond merely describing atypical segmentation behavior in ASD, we aimed to delineate the underlying cognitive processes that differ from those of their NT peers. Moreover, we aimed to separately examine processes leading up to the closure of an event segment and processes related to establishing a new event segment. To this end, we used neurophysiological data, since processes described in EST can be distinctly associated with oscillatory activity in the EEG alpha, beta and theta frequency bands (Prochnow et al., 2024b). Alpha band activity (ABA) in inferior parietal regions is related to gating access to memory (Klimesch, 2011, Klimesch, 2012), so that reduced ABA at event boundaries likely represents facilitated access to event schemata for updating the working event model (Ghorbani et al., 2024, Prochnow et al., 2024b). Furthermore, setting an event boundary is associated with attenuated prefrontal beta band activity (BBA). BBA is linked to the stable maintenance of working memory representations (Engel and Fries, 2010, Spitzer and Haegens, 2017) and hence decreases when working event models are updated (Prochnow et al., 2024b). Lastly, theta band activity (TBA) was found to subserve an error signaling function (Cavanagh et al., 2012a). Thus, when working event models no longer allow an accurate prediction of upcoming events and have to be updated, TBA in mid-frontal regions increases (Prochnow et al., 2024b). Since individuals with ASD are thought to rely less on event schemata (Goris et al., 2018, Van de Cruys et al., 2017), parietal ABA modulations at event boundaries are expected to be lower for ASD than NT participants. Due to a greater susceptibility to sensory input fluctuations resulting in a relative instability of working event models, we also assume prefrontal BBA modulations at event boundaries to be attenuated in ASD adolescents. Lastly, if prediction errors are weighted more heavily in ASD participants, more pronounced mid-frontal TBA increases at event boundaries can be anticipated. These modulations were assumed to differ in both their magnitude and their spatial distribution.

## 2 Materials and methods

### 2.1 Participants

We collected data from N = 36 adolescents diagnosed with high functional ASD and N = 90 NT adolescents. The larger pool of neurotypical subjects has also been examined in other publications (Ghorbani et al., 2024, Prochnow et al., 2024a, Zhou et al., 2025). Exclusion criteria for NT participants included below-average IQ and diagnosis of neurological or psychiatric disorders. For ASD participants, below-average IQ, neurological disorders, and certain comorbid disorders including obsessive–compulsive-disorder, attachment disorder, and tic disorder were reasons for exclusion. Medication intake in the ASD group was allowed only if the medication was methylphenidate-based and the last time of intake occurred at least 6 h prior to the study appointment, given methylphenidate’s reported duration of action of 1 to 4 h (Kimko et al., 1999). All patients with ASD were diagnosed applying the ICD-10 diagnostic system by trained clinicians independent of the study. Under consideration of the German national guideline (S3-Leitlinie), diagnosis of ASD was ascertained using the Autism Diagnostic Interview “ADI-R” (Bölte et al., 2006, Lord et al., 1994), the German version of the Autism Diagnostic Observation Schedule “ADOS-2” (Poustka et al., 2015), and several ASD-specific questionnaires such as the social responsiveness scale (Bölte and Poustka, 2007), social communication questionnaire (Bölte and Poustka, 2006) and the Marburger Evaluation scale for Aspergers Syndrome (Kamp-Becker et al., 2005). Written informed consent was obtained from all participants and their legal guardians before any study procedure was applied. The local ethics committee of the Medical Faculty of the TU Dresden approved the study (ethics reference number: SR + BO-EK-495112020, approval date: February 10th 2022). The study was conducted in strict accordance with institutional guidelines and data protection standards, ensuring that participants’ privacy rights were respected at all stages of data collection and processing. From the initial ASD sample, we had to exclude N = 2 participants due to the intake of medication shortly before the appointment, and N = 2 additional participants were excluded due to technical issues. We used the remaining N = 32 participants in the ASD group as a reference for a subsequent group-matching procedure to select an equal number of NT adolescents for comparison. Matching was conducted on the basis of participants’ age and sex assigned at birth by application of an optimal pair matching algorithm using the MatchIt() function (D. Ho et al., 2011) in R version 4.4.1. This method pairs similar individuals so that the distance in covariate space is minimized across all matches (Gu and Rosenbaum, 1993; D. E. Ho et al., 2007). During the processing of behavioral data, N = 1 subject from the ASD group was determined as an outlier as their number of responses was three SD above the sample mean, and hence was excluded. EEG preprocessing resulted in a further reduction in sample size, with N = 1 NT participant being excluded as more than a third of their channels required interpolation following the removal of bad channels (see section 2.3). Finally, N = 1 ASD participant and N = 1 NT participant were excluded as the preprocessed EEG data contained no valid responses (see section 2.3). Consequently, the final sample size was N = 60 participants with N = 30 subjects in the ASD group (13.5 ± 1.7 years, 28 male and 2 female according to sex assigned at birth) and N = 30 subjects in the NT group (13.5 ± 1.8 years, 28 male and 2 female according to sex assigned at birth). In the ASD group, none of the final participants was medicated. An overview of all comorbid psychiatric disorders present in the final ASD sample is provided in Supplemental Table 1.

The final groups did not differ in terms of age (*t*(58) = 0.07, *p* = 0.942). Similarly, there were no group differences in the two subtests of the IQ Screening from the Intelligence and Developmental Scales 2 (IDS-2) (Grob and Hagmann-von Arx, 2018), matrices completion and category naming (Tab. 1). Before the appointment, parents reported on their children’s current psychopathological symptom status (see Tab. 1 for descriptive statistics and group comparisons) by completing the German versions of the Child Behavior Checklist (CBCL/4–18) (Döpfner et al., 2014) and the Social Communication Questionnaire (SCQ) (Bölte and Poustka, 2006). Parent questionnaire data for the Child Behavior Checklist (CBCL/4–18) (Döpfner et al., 2014) were missing from N = 10 ASD participants and N = 1 NT participant, and for the Social Communication Questionnaire (SCQ) (Bölte and Poustka, 2006) from N = 8 ASD participants and N = 1 NT participant. With respect to the available questionnaires, evaluation of the CBCL revealed that ASD participants scored higher than the NT participants on all but one (rule breaking behavior) syndrome scales (see Tab. 1). Similarly, across all SCQ subscales, impairments of social communicative skills were more pronounced for the ASD group, than for NT adolescents (see Tab. 1). Parents’ educational levels, as reported in a brief questionnaire, differed between groups (χ2 (4, 105) = 10.47, *p* = 0.033). In the NT group, 15.0% of parents reported having obtained a doctoral degree, 43.3% were university graduates, 20.0% had graduated from college, 20.0% had completed 10 years of formal education, and 1.7% had not provided information on their education. In the ASD group, 3.3% of parents reported to have obtained a doctoral degree, 28.3% were university graduates, 10.0% had obtained a college degree, 31.7% had completed 10 years of formal education, 3.3% had finished school after 9 years of formal education, and 23.3% provided no information on their educational level. Hence, parents in the NT group reported overall higher educational levels.

**Table 1:** Descriptive statistics and *t*-test results for the group comparison of IQ-dimensions and parent questionnaire data in the SCQ and CBCL.

	**NT: M(SD)**	**ASD: M(SD)**	**df**	***t***	***p***
IQ dimensions					
MC	96.7 (14.5)	101.5 (16.7)	58	−1.20	0.237
CN	108.7 (12.9)	104.8 (19.5)	58	0.90	0.372
SCQ					
Total	3.8 (2.4)	18.7 (8.1)	49	−9.41	< 0.001
SocInt	1.0 (0.9)	7.7 (4.0)	49	−8.82	< 0.001
Com	1.9 (1.9)	6.5 (2.8)	49	−6.92	< 0.001
SteBeh	0.8 (1.3)	4.1 (2.6)	49	−6.10	< 0.001
CBCL					
SW	51.8 (4.3)	62.9 (9.6)	47	−5.51	< 0.001
SC	53.1 (4.0)	56.5 (6.8)	47	−2.19	0.034
AD	51.7 (3.9)	56.7 (7.7)	47	−2.98	0.005
SP	50.8 (3.4)	66.0 (8.4)	47	−8.71	< 0.001
TP	51.0 (2.7)	65.1 (11.9)	47	−6.15	< 0.001
AP	51.3 (3.5)	63.0 (8.6)	47	−6.54	< 0.001
RB	52.3 (5.5)	55.6 (6.3)	47	−1.95	0.059
AB	51.0 (3.6)	58.4 (7.9)	47	−4.39	< 0.001

MC = matrices completion, CN = category naming, Total = total SCQ score; SocInt = social interaction; Com = communication; SteBeh = stereotypical behavior. SW = social withdrawal; SC = somatic complaints; AD = anxious/ depressed; SP = social problems; TP = thought problems; AP = attention problems; RB = rule breaking behavior; AB = aggressive behavior. Given are the mean of the t-scores with standard deviations in parentheses. Tests on SCQ values were Bonferroni-corrected for 4 multiple comparisons.

### 2.2 Task

Event segmentation was assessed using an established paradigm (Zacks et al., 2009) in which participants watch the short movie “The Red Balloon” (30 min, no audio track) (Lamorisse, 1956) and are asked to divide it into discrete events. In order to familiarize themselves with the task, participants first completed a practice session using a short video (5 min) showing a man decorating a room for a party (Sargent et al., 2013). This was followed by the main task in which “The Red Balloon” movie was divided into three clips (10 min each) interleaved by breaks of self-adjustable length. The movie was presented using the software “Presentation” (RRID:SCR_002521), which also recorded participants’ button press responses. Participants were instructed to press the space bar whenever they felt that a meaningful unit (i.e., an event) had ended and a new one had begun (Newtson and Engquist, 1976). For the analysis of event segmentation behavior, we divided the movie into consecutive 2 sec time bins, ensuring a reasonable balance between temporal resolution and analytic feasibility. Given the frame rate of 30 frames per second, each bin comprised 60 individual frames. The occurrence of button presses within each bin served as the dependent variable. To examine the relationship between subjectively perceived event boundaries (button presses) and objective changes in the movie scene, we drew on the situational change coding developed by Zacks et al. (2009). This coding is based on a frame-by-frame analysis and distinguishes nine types of situational changes, namely character changes (focus switches from one character to another), character-character changes (regards the interplay between characters), character-object changes (shifts in the dynamics between a character and an object), temporal changes (jumps in time), large spatial changes (modifications in camera perspective), small spatial changes (alterations in a character’s location), changes in cause (events in the current frame are not a result of some activity from the previous frame), goal changes (shifts in a character’s goal) and scene changes (editorial cuts).

### 2.3 EEG Recording and preprocessing

Continuous EEG data were recorded at the University Hospital Dresden by utilizing BrainAmp Amplifier and Brain Vision Recorder 1.2 software (Brain Products, Gilching, Germany) and elastic caps (Easycap, Wörthsee, Germany) with 60 built-in Ag-AgCl electrodes in an equidistant layout (reference electrode at FPz; ground electrode at θ = 58, φ = 78). Electrode impedances were kept below 5 kΩ. Data were initially collected at a sampling rate of 500 Hz and later downsampled offline to 300 Hz to match the frame rate of the RedBalloon movie (Lamorisse, 1956). For preprocessing, the ‘automagic’ pipeline (Pedroni et al., 2019) and EEGLAB (Delorme and Makeig, 2004) were employed in MATLAB R2022a (The MathWorks Corp, RRID:SCR_001622), similar to a recent pipeline by our group (Böttcher et al., 2026). At first, 50 Hz line noise was removed by means of a multitaper algorithm provided in PREP (Bigdely-Shamlo et al., 2015), before data was re-referenced by application of a robust average reference based on all good channels. Subsequently, to detrend the EEG data, a FIR high-pass filter of 0.5 Hz (order 1286, stop-band attenuation 80 dB, transition band 0.25 – 0.75 Hz) as contained in the EEGLAB clean_rawdata() function was applied. Additionally, this function also eliminates bad channels that are flatlined (absence of signal variation for more than 5 sec), show low correlation with other channels, or have high variation over time (standard deviation > 75 μV). Furthermore, clean_rawdata() allows to discern time windows with heightened noise bursts and utilizes subspace statistics to reconstruct signal components that have been corrupted by using the Artifact Subspace Reconstruction (ASR; burst criterion: 15) method (Mullen et al., 2013). If time windows could not be reconstructed, they were removed. Subsequently, data were passed through a 40 Hz low-frequency filter (sinc FIR filter; order: 86) (Widmann et al., 2015). Further cleaning of data entailed the application of a subtraction method (Parra et al., 2005) to separate EOG artefacts, as well as the employment of the Multiple Artifact Rejection Algorithm (MARA) (Winkler et al., 2011, Winkler et al., 2014) that performs an independent component analysis and rejects those components that are classified as corresponding to muscle, heart or eye movement artefacts. Eventually, a spherical method was used to interpolate all missing or eliminated channels. A comparison between the NT and ASD group with respect to EEG data quality metrics (i.e. number of interpolated channels and omitted noisy segments) is provided in Supplemental Analysis 1.

To identify time windows of interest, we partitioned the EEG data into 2 sec segments using the FieldTrip toolbox (Oostenveld et al., 2011). For each participant separately, we then determined which segments contained a response that indicated an event boundary (Boundary intervals; BI) and which segments did not (No-Boundary intervals; NBI). In the case of two adjacent responses occurring with fewer than 4 sec delay to each other, we omitted the second response to prevent overlapping segments. Across all participants, we found this procedure to result in a greater number of NBI than BI. Thus, for each of the three movie clips, we then randomly selected as many samples from the NBI as there were BI within the same clip and sorted both interval types according to their occurrence in the movie. This resulted in a pairing of BI and NBI occurring around a comparable time and ensured that EEG noise levels and participants’ levels of fatigue were similar in contrasted intervals. Subsequently, we determined the exact location of response triggers within BI and inserted a virtual response marker at the same time point into the corresponding NBI. Lastly, we realigned the 2 sec data segments so that the response or virtual marker was centered within a segment. Accordingly, our analyzed EEG segments encompass a time window of −1 sec to + 1 sec around the response or virtual markers. The reason for us following this response locking approach lies in the unique nature of the used paradigm, which, for one, makes use of continuous stimulus material. For another, the time point of event boundary perception, as indicated by participants’ responses, is the most central determinant to retrace the event segmentation process.

### 2.4 Beamforming and source level analysis of oscillatory activity

All oscillatory activity analyses were conducted independently for the pre-response and post-response time windows using the FieldTrip toolbox (Oostenveld et al., 2011). We focused the analyses on three frequency bands of interest: theta (4–7 Hz), alpha (8–12 Hz) and beta (15–30 Hz) band activity. A sensor-level comparison between conditions and groups employing a wavelet-based time–frequency analysis (Morlet wavelet) is described in Supplemental Analysis 2. Reconstruction of the oscillatory activity sources was done by applying the dynamic imaging of coherent sources (DICS) beamforming method (Gross et al., 2001) with a rank reduction of 3 (default for EEG) and the regularization parameter lambda set to 5%. The DICS beamforming method entailed the following steps: First, a spatial filter for each participant and each time window (pre-boundary and post-boundary) is created by appending data from the boundary and no-boundary conditions and computing the cross-spectral density matrix obtained from applying a Hanning taper with a smoothing box of 0.5 Hz to the activity in frequencies of interest. This common spatial filter is applied across conditions within the same subject. Using the boundary element volume conduction model provided in FieldTrip (Oostenveld et al., 2011), activity is then projected onto a 3D model of the brain that is expressed in the Montreal Neurological Institute (MNI) coordinate system and divided into a grid with 0.5 cm resolution (constituting voxels of the size 0.5*0.5*0.5 cm).

Consistent with the sensor-level analysis (see Supplemental Analysis 2), we subsequently identified frequency bands associated with neural activity related to event boundary detection by conducting a within-group cluster-based permutation test (CBPT; 1 000 permutations; clustering performed across space, time, and the range of frequencies constituting a frequency band of interest; *p*-value corrected at cluster-level). This analysis compared average source activity between the BI and NBI conditions across all voxels for each frequency band. Frequency bands that did not show significant condition differences were not included in the subsequent group comparisons. To enable a between group comparison, we then repeated the previous CBPT in a leave-one-out-approach (Prochnow et al., 2025). As our groups contained 30 participants each, this procedure entailed 30 iterations per group. In the first iteration we computed and compared the average absolute power estimate per condition and frequency band based on data from all but the first group member, in the second iteration we dismissed data from the second group member and so on. As a result, we obtained as many sets of CBPT statistics as there were participants within each group. To compare these results across groups, we conducted Wilcoxon-Mann-Whitney tests (using the *wilcox_test* function in R), and calculated corresponding effect sizes (r) with the *rstatix* package (Kassambara, 2023). Group comparisons were based on two metrics: the size of significant clusters (i.e., the number of significant voxels) and the relative modulation strength, relT_sum (i.e., the size of absolute power differences between BI and NBI in terms of $T_{sum}$ divided by the number of voxels comprising that cluster).

Thereafter, we localized the neuroanatomical structures showing the strongest contrast between the BI and NBI conditions using the Density Based Spatial Clustering of Applications with Noise (DBSCAN) (Ester et al., 1996). DBSCAN was configured to identify clusters of spatially coherent voxels exhibiting the top 1% of activity differences between conditions, while disregarding voxels belonging to the cerebellum. The search radius for adjacent significant voxels was 0.75 cm (epsilon = 1.5*gridsize) and a minimum of five voxel had to group together to be considered a cluster. The identified voxels were then mapped to neuroanatomical regions using the Automatic Anatomical Labeling (AAL) atlas (Tzourio-Mazoyer et al., 2002).

### 2.5 Statistics

For the statistical analysis of behavioral data, Bayesian estimation of mixed-effects logistic regression was conducted in R version 4.4.1 (R Core Team), using the brms function (Bürkner, 2017, Bürkner, 2018) that is based on a Markov chain Monte Carlo (MCMC) method. Separate models were fitted to investigate 1) how the number of changes occurring within a 2 sec interval affects participants’ likelihood to set an event boundary and 2) to determine the influence of different types of situational changes on participants’ segmentation probability. In all of our models, a random intercept on the subject level was included to account for interindividual variability. For 1), the total number of changes per interval (possible values: 0–5) was counted. This factor, alongside the information to which group a participant belonged (NT = 0 vs ASD = 1), was then entered into the model as a predictor for the binary outcome of whether participants had indicated an event boundary for a certain interval or not (occurrence = 1 vs absence = 0). In 2), we used the same outcome as in 1), but modeled its dependency on the occurrence (1) or non-occurrence (0) of each of the nine types of situational change within a 2 sec segment, as well as on participants’ group membership. For both analyses, we report credible intervals (CrI) as the Bayesian indicator of estimate uncertainty akin to confidence intervals (CI) in the frequentist framework (Hespanhol et al., 2019). Moreover, Odds Ratios (OR) as a measure of effect size were computed by exponentiating the Bayesian model estimates. An independent *t*-test was performed to compare groups regarding the mean length of their segmented events (calculated by dividing the duration of the movie by the number of responses).

To account for the possibility that disliking the movie might reduce task engagement, we compared Likert scale ratings of how much NT and ASD participants enjoyed the RedBalloon movie by performing a Wilcoxon rank sum test (Supplemental Analysis 3). Further, we calculated point-biserial correlations to quantify the agreement between NT and ASD participants with regard to the timing of identified event boundaries across the movie (Supplemental Analysis 4).

## 3 Results

### 3.1 Behavioral results

**Fig. 1:**
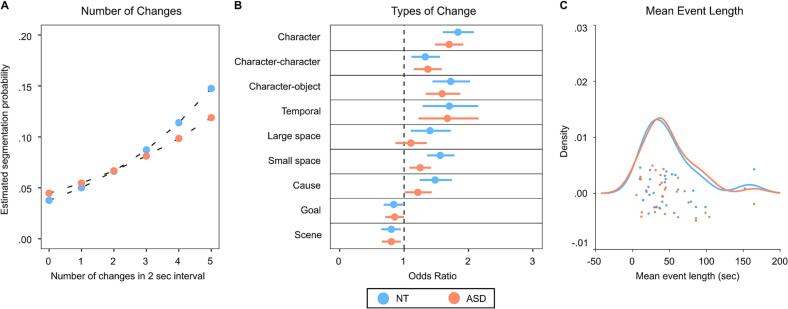
Behavioral results. (A) Results of the Bayesian Logistic Regression that estimated how participants’ segmentation probability (y-axis) changed as a function of the number of changes taking place within a 2 sec movie interval (x-axis). (B) Results of the Bayesian Logistic Regression that assessed the influence of different types of situational changes (y-axis) on the probability of segmentation (expressed in Odds Ratios, x-axis). Dots designate mean Odds Ratios per group, while error bars signify the 95% confidence interval. (C) Density distribution for the mean length of segmented events. Dots represent single participants’ values. Values for ASD patients are presented in orange and for NT participants in blue. (For interpretation of the references to colour in this figure legend, the reader is referred to the web version of this article.)

The estimation of segmentation likelihood dependent on the number of changes per interval and group membership (intercept: *β* = -3.26 ± 0.16, 95% CrI [-3.59, −2.94], OR = 0.04, 95% Cl [0.03, 0.05]) revealed a significant effect for the total number of changes (*β* = 0.30 ± 0.02, 95% CrI [0.26, 0.33], OR = 1.35, 95% Cl [1.29, 1.40]). The more situational changes occurred within a segment, the more likely participants were to set an event boundary for this segment (Fig. 1A).

Yet, this main effect was qualified by a significant interaction with participants’ group membership (*β* = -0.09 ± 0.03, 95% CrI [-0.14, −0.03], OR = 0.92, 95% Cl [0.87, 0.97]). Compared to NT participants (*β* = 0.30 ± 0.02, 95% CrI [0.26, 0.33], OR = 1.35, 95% Cl [1.30, 1.40]), an increment in the number of changes per segment resulted in a less steep increase in segmentation likelihood in the ASD group (*β* = 0.21 ± 0.02, 95% CrI [0.17, 0.25], OR = 1.24, 95% Cl [1.19, 1.28]). Withal, group membership alone did not elicit a substantial effect on participants’ segmentation probability (*β* = 0.19 ± 0.23, 95% CrI [-0.26, 0.62], OR = 1.21, 95% Cl [0.77, 1.86]).

In the regression model probing the predictive value of different types of situational changes and group membership for segmentation behavior (intercept: *β* = -3.21 ± 0.11, 95% CrI [-3.44, −2.99], OR = 0.04, 95% Cl [0.03, 0.05]), displayed in Fig. 1B, an interaction between group membership and the occurrence of Small Space changes appeared (*β* = -0.22 ± 0.09, 95% CrI [-0.40, −0.04], OR = 0.80, 95% Cl [0.67, 0.96]). When determining event boundaries, adolescents from the ASD group (*β* = 0.22 ± 0.07, 95% CrI [0.08, 0.35], OR = 1.24, 95% Cl [1.09, 1.41]) used changes in the movement of characters to a lesser extent than NT adolescents did (*β* = 0.44 ± 0.07, 95% CrI [0.31, 0.57], OR = 1.55, 95% Cl [1.36, 1.78]). Other interactions between types of situational changes and group membership were not observed (for complete model estimates see Suppl. Tab. 4).

Convergence diagnostics (i.e., R-hat and effective sample sizes; see Suppl. Tab. 5 and 6) and posterior predictive tests (see Suppl. Figs. 2 and 3) for the Bayesian logistic regression models are provided in the Supplemental material.

The groups did not differ with regard to the mean length of indicated events (*t*(58) = 0.67, *p* = 0.503), Fig. 1C.

### 3.2 Neurophysiological results on source level before boundaries

**Fig. 2:**
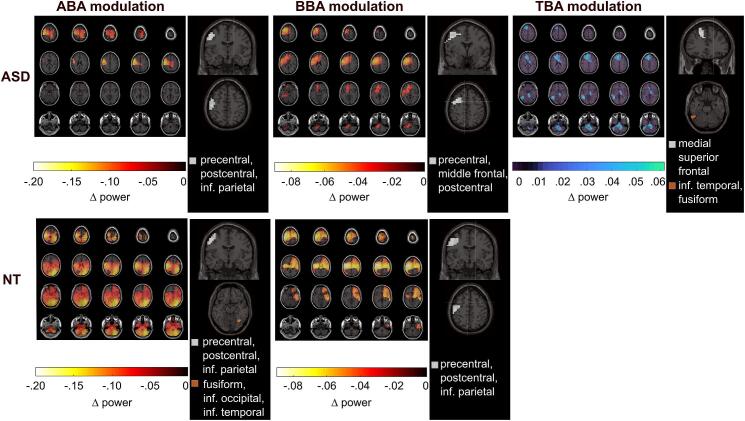
Source level neurophysiological results for the pre-boundary period. In each column (left: ABA, middle: BBA, right: TBA), the left plot displays the average power difference between BI and NBI intervals in the significant voxels identified through cluster-based permutation testing. In the right plot of each column, voxels exhibiting the top 1% activity difference (BI minus NBI) are accentuated. Different colors are used to mark separate clusters. Results for ASD participants are shown in the first row and for NT participants in the second row. Depiction of TBA modulations in NT participants is omitted as significant clusters for the BI – NBI contrast were absent.

Tab. 2 displays the cluster statistics of pre-boundary and post-boundary source level analysis. Source-level activity differences between BI and NBI for TBA were not significant in the NT group (23 positive clusters: *p* ≥ 0.063, one negative cluster: *p* = 0.701), but in the ASD group (among 40 positive clusters, one was significant). As no iteration of the leave-one-out CBPT produced a TBA cluster in the NT group, no between-group comparison of TBA modulation was conducted. The DBSCAN algorithm identified two left-lateralized clusters as the source of the strongest TBA modulations in ASD, one comprising the inferior temporal region (BA20) and the fusiform gyrus (BA37), and the second cluster containing the superior frontal gyrus (BA6/BA8/BA9), including its medial subdivision, Fig. 2.

**Table 2:** Cluster statistics for all significant clusters observed in the CBPT contrasting oscillatory activity between BI and NBI on source level.

		**NT**		**ASD**	
**Time window**	**Frequency Band**	**T_sum_**	***p***	**T_sum_**	***p***
Pre-response	TBA	/	/	3 913.77	0.034
Pre-response	ABA	−42 720.15	0.001	−6 544.76	0.033
Pre-response	BBA	−14 930.70	0.001	−9 232.68	0.010
Post-response	TBA	−4 614.72	0.021	/	/
Post-response	ABA	−58 681.57	0.001	−41 390.32	0.001
Post-response	BBA	−5 894.49	0.023	/	/

TBA = theta band activity, ABA = alpha band activity, BBA = beta band activity.

Regarding ABA, NT and ASD participants alike showed a relative down-modulation of ABA during BI compared to NBI (NT: among 32 negative clusters one significant, ASD: among 49 negative clusters one significant). In NT participants, regions associated with the highest ABA modulation were localized in one right-lateralized cluster consisting of the fusiform gyrus (BA37), inferior occipital gyrus (BA18/BA19) and inferior temporal cortex (BA20) and one left-lateralized cluster made up by the precentral (BA4) and postcentral gyrus (BA1/BA2/BA3), inferior parietal cortex (BA39/BA40) and supramarginal gyrus (BA40). A similar left-lateralized cluster involving the precentral (BA4) and postcentral gyrus (BA1/BA2/BA3), inferior parietal cortex (BA39/BA40) and supramarginal gyrus (BA40) was identified in ASD participants. Between-group comparison of ABA modulation using the leave-one-out approach revealed the cluster size (Z = 6.85, p < 0.001, r = 0.88) and the relative strength of ABA modulation between conditions (relT_sum_) (Z = -6.77, p < 0.001, r = 0.87) to be smaller in ASD compared to NT participants.

For BBA, a relative down-modulation during BI compared to NBI was evident in NT and ASD participants (NT: among 74 negative clusters one significant; ASD: among 54 negative clusters one significant). The DBSCAN localized highest BBA modulations in NT in a left-hemispheric cluster containing the precentral (BA4) and postcentral gyrus (BA1/BA2/BA3), inferior parietal cortex (BA39/BA40) and supramarginal gyrus (BA40). Strongest BBA modulations in ASD were linked to the left-hemispheric precentral (BA4) and postcentral gyrus (BA1/BA2/BA3), middle frontal gyrus (BA9), superior frontal gyrus (BA6/BA8/BA9) and supramarginal gyrus (BA40). The leave-one-out CBPT revealed the cluster size in BBA to be smaller in ASD than NT participants (Z = 6.21, *p* < 0.001, *r* = 0.80), yet, the relative strength of modulation was higher in ASD participants (Z = 6.65, *p* < 0.001, *r* = 0.86).

To test the robustness of the identified regions of greatest activity modulations, we performed a sensitivity analysis with alternative DBSCAN thresholding levels (i.e. 0.5% and 2%). Results of this analysis for the pre-boundary period are provided in Supplemental Table 7 and 8 as well as in Supplemental Fig. 4.

### 3.3 Neurophysiological results on source level after boundaries

**Fig. 3:**
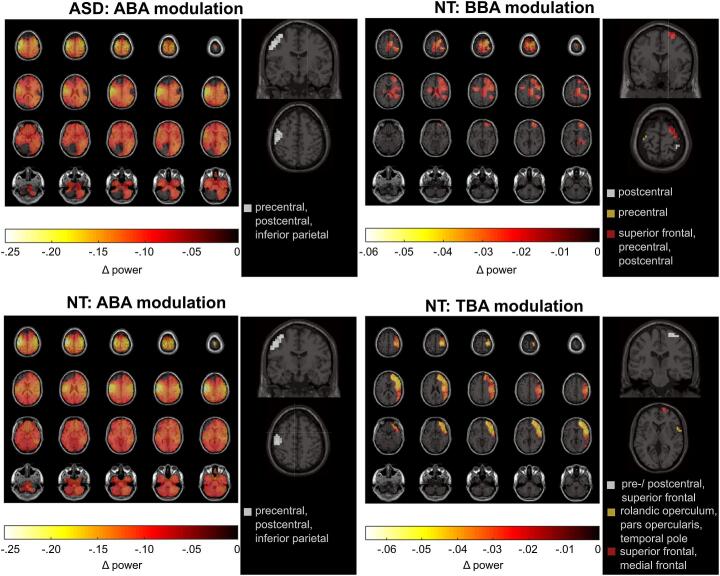
Source level neurophysiological results for the post-boundary period. In each column the left plot displays the average power difference between BI and NBI intervals in the significant voxels identified through cluster-based permutation testing. In the right plot of each column, voxels exhibiting the top 1% activity difference (BI minus NBI) are highlighted. Each independent cluster is assigned a different color. The first column shows results for ABA modulations in ASD (first row) and NT participants (second row), whereas the second column displays BBA (first row) and TBA (second row) modulations in NT participants. Depiction of BBA and TBA modulations in ASD participants are omitted as there were no significant clusters for the BI – NBI contrast.

The CBPT examining oscillatory activity differences between BI and NBI revealed a non-significant positive TBA cluster ($T_{\mathrm{sum}}$ = -451.47, *p* = 0.265) and a significant negative TBA cluster in the NT group (among 82 negative clusters one significant), but no significant modulation in the ASD group (16 positive clusters: *p* ≥ 0.116; 26 negative clusters: *p* ≥ 0.246; Fig. 3). Given that no iteration of the leave-one-out approach generated a significant TBA cluster in ASD, we did not compare the TBA modulation between groups. The neuroanatomical origins of greatest TBA modulations in the NT group were dispersed across three right-lateralized clusters: First cluster containing the superior frontal gyrus (BA6/BA8/BA9) including its medial and orbital parts and the orbital part of the medial frontal gyrus (BA9/BA46), second cluster including the Rolandic operculum (part of BA43), the inferior frontal gyrus pars opercularis (BA44) and the superior part of the temporal pole (BA38), and third cluster encompassing the precentral (BA4) and postcentral gyrus (BA1/BA2/BA3), superior frontal gyrus (BA6/BA8/BA9) and the supplementary motor area (part of BA6).

Lower ABA during BI than NBI was evident in NT (among 15 negative clusters, one significant) and ASD participants (among 40 negative clusters, one significant). DBSCAN results suggested a left-lateralized localization of the largest modulation, with slight group differences emerging with regard to the neuroanatomical structures constituting the clusters: In NT, regions with the greatest BI minus NBI activity difference included the precentral gyrus (BA4), postcentral gyrus (BA1/BA2/BA3), inferior parietal regions (BA39/BA40), the supra-marginal gyrus (BA40) and superior frontal gyrus (BA6/BA8/BA9), whereas in ASD the cluster additionally comprised middle frontal regions (BA8/BA9). As indicated by the leave-on-out CBPT, the size of the cluster associated with this ABA modulation (Z = 6.61, *p* < 0.001, *r* = 0.85) and the magnitude of ABA modulation (relT_sum_) (Z = -6.65, *p* < 0.001, *r* = 0.86) were smaller in ASD compared to NT participants.

Condition-specific BBA modulations were limited to the NT group, which exhibited four non-significant positive clusters (p ≥ 0.758) and one significant cluster among 26 negative clusters, whereas no significant clusters emerged in the ASD group (three positive clusters: *p* ≥ 0.653; 13 negative clusters: *p* ≥ 0.163). Within the NT group, the greatest BBA down-modulation during BI relative to NBI was observable in three distinct clusters: The first right-lateralized cluster encompassed the superior frontal gyrus (BA6/BA8/BA9) as well as the precentral (BA4) and postcentral gyrus (BA1/BA2/BA3). The second cluster was ascribed to the left precentral gyrus (BA1/BA2/BA3) and the third cluster to the right postcentral gyrus (BA1/BA2/BA3). Between-group comparison of BBA modulation using the leave-one-out approach revealed the cluster size (*Z* = 4.13, *p* < 0.001, r = 0.53) and the relative strength of modulation between conditions (relT_sum_; Z = -4.08, p < 0.001, r = 0.53) to be smaller in ASD compared to NT participants.

The robustness of the observed neuroanatomical origins of greatest activity modulations even at alternative DBSCAN thresholding levels is illustrated in Supplemental Table 9 and 10 as well as in Supplemental Fig. 5.

## 4 Discussion

In the present study, we assessed differences in event segmentation between adolescents with ASD and their neurotypical peers to explore this cognitive process as a potential mechanism underlying ASD symptomatology. Event segmentation (Zacks, 2020, Zacks and Swallow, 2007) was measured by asking participants to parse a movie into discrete, meaningful units. Additionally, we investigated neurophysiological indicators associated with the EST components by examining modulations of ABA, BBA, and TBA at event boundaries and their neuroanatomical localization.

We found that segmentation behavior in adolescents with ASD was less related to situational changes compared to neurotypical peers, consistent with accounts of global processing difficulties in ASD (Happé and Frith, 2006, Plaisted, 2001). Using a task simulating complex natural events, our findings extend previous research with abstract paradigms and support the view that individuals with ASD struggle when tasks require dynamic integration of sensory signals over time (Robertson and Baron-Cohen, 2017). Notably, our data do not support the idea of general detail sensitivity or heightened prediction error weighting in ASD (Mottron et al., 2006, Van de Cruys et al., 2017), as no group differences emerged in the mean event length. Instead, adolescents with ASD showed reduced consideration of small spatial changes (i.e. changes in characters’ movements), aligning with evidence of difficulties in perceiving biological motion in complex contexts (Todorova et al., 2019). In contrast, despite frequent reports of impaired social cue processing in abstract tasks (Jellema et al., 2009, Robic et al., 2015), the ASD group effectively used social information (character and interaction changes) to structure the movie, likely because these cues were contextualized and salient. This highlights the value of naturalistic paradigms to reveal how isolated deficits may be compensated under everyday conditions.

The neurophysiological analysis revealed distinct roles of TBA modulations before event boundaries. Adolescents with ASD showed increased left midfrontal TBA prior to boundaries, similar to adults (Prochnow et al., 2024b), consistent with its role in conflict monitoring and error signaling (Cavanagh et al., 2012a, Cavanagh and Frank, 2014, van Driel et al., 2012) and with EST assumptions about discrepancies driving boundary setting (Kurby and Zacks, 2008, Zacks et al., 2007, Zacks and Sargent, 2010). Additional pre-boundary TBA upregulation in ASD was evident on the source-level and associated to the left inferior temporal and fusiform gyri, regions linked to visual sequence violations (Esmailpour et al., 2023) and social information processing (Iacoboni et al., 2004, Lee Masson and Isik, 2021, Prochnow et al., 2024a). This may reflect detection of deviations despite absent behavioral effects (Lawson et al., 2017). Notably, NT participants lacked significant pre-boundary TBA modulation on the source-level, suggesting error signaling is not required for their boundary perception. Group differences also emerged in ABA down-modulation. NT participants, but not ASD, showed a right fusiform cluster, linked to non-verbal semantic memory (Mion et al., 2010) and event schema access (Prochnow et al., 2024b), in addition to down-modulations in postcentral and parietal regions reflecting episodic retrieval (Prochnow et al., 2024b). In ASD, ABA reductions were limited to postcentral/parietal areas, consistent with deficient use of global event schemata (Goris et al., 2018). For BBA, both groups showed the strongest down-modulation in left pre- and postcentral gyri, indexing event model updating (Prochnow et al., 2024b), though motor preparation cannot be excluded. In ASD, BBA modulation was more focal but of greater magnitude, suggesting compensatory overactivation. Overall, boundary setting in ASD relied on increased error monitoring, whereas neurotypicals strengthened event schema access.

After event boundaries, NT participants − but not ASD − showed TBA down-regulations in the right Rolandic operculum, inferior and superior frontal regions, linked to cognitive control and performance monitoring (Beste et al., 2023, Cavanagh et al., 2012b, Cavanagh and Frank, 2014, Dippel and Beste, 2015, Prochnow et al., 2024b). This likely reflects a temporary reduction of monitoring to allow the unhindered formation of a new event segment. In ASD, this attenuation is absent, possibly impeding valid event model formation. For ABA, both groups showed similar patterns, though ASD exhibited more focal and weaker down-modulation, suggesting continued episodic schema retrieval at the start of new segments (Prochnow et al., 2024b). Crucially, only NT participants displayed post-boundary BBA down-modulation in right superior frontal regions, associated with updating working event models (Engel and Fries, 2010, Prochnow et al., 2024b, Spitzer and Haegens, 2017). This effect is unlikely to be motor-related, as participants responded with the right index finger, but modulation occurred ipsilaterally.

Overall, NT participants seem to recruit multiple processes to establish new event segments, whereas adolescents with ASD rely mainly on weaker schema retrieval. Of note, these differences on the neurophysiological level were only observed on the source level, while measurements at the sensor level did not establish any group differences (see Supplemental Analysis 2). This aligns with recent findings demonstrating that error detection is not the only way to trigger an event boundary; contextual transitions stored in event schemata can also initiate event segmentation (Güler et al., 2024, Nolden et al., 2024, Wang et al., 2024). Upon the establishment of a new event segment, performance and error monitoring are reduced, while the updating process is amplified and a simultaneous retrieval of event schemata occurs. In contrast, in patients with ASD the closing of an event segment is characterized by increased error detection, retrieval of event schemata, and the initiation of working event model updating. After the event boundary is set, however, only the retrieval of event schemata seems to remain active. Thus, in ASD, several processes occur during the closing of an event segment, but the opening of the new segment receives comparatively less emphasis.

In conclusion, event segmentation in adolescents with ASD is characterized by an altered balance of error monitoring and schema use. Unlike NT peers, who rely on anticipatory, schema-driven segmentation, adolescents with ASD emphasize error detection and show reduced updating when forming new segments. This atypical modulation of control and memory processes may underlie reduced flexibility in event perception. Importantly, these alterations were observed in a high-functioning ASD sample with comparatively preserved cognitive abilities, suggesting that event segmentation may represent a particularly vulnerable cognitive function. While the relative homogeneity of our sample with respect to IQ and functionality constraints the generalizability of these conclusions to the broader autism spectrum, the broad range of comorbidities increases representativeness for patient cohorts presenting with a range of psychiatric symptoms. Nevertheless, future research should systematically examine event segmentation across a wider range of ASD symptom severity levels to determine the robustness of these effects. Such work would be particularly informative if combined with assessments of the real-world impact of event segmentation alterations or with intervention-based approaches probing the malleability of event segmentation through targeted interventions (e.g., training of predictive processing or schema use).

## CRediT authorship contribution statement

**Ronja Limburg:** Writing – original draft, Visualization, Validation, Software, Investigation, Formal analysis, Data curation. **Michel Benjamin Kopp:** Writing – original draft, Formal analysis, Data curation. **Xianzhen Zhou:** Writing – original draft, Software, Investigation. **Foroogh Ghorbani:** Writing – original draft, Visualization, Investigation. **Veit Roessner:** Writing – review & editing, Resources, Funding acquisition, Conceptualization. **Bernhard Hommel:** Writing – review & editing, Funding acquisition, Conceptualization. **Christian Beste:** Writing – review & editing, Supervision, Resources, Project administration, Funding acquisition, Conceptualization. **Astrid Prochnow:** Writing – review & editing, Visualization, Validation, Supervision, Methodology, Investigation, Data curation.

## Funding

This work was supported by a grant from the Else-Kröner Fresenius Stiftung (Key project) to CB, BH, and VR (2020_EKSE.105), a grant from the Else-Kröner Fresenius Stiftung to AP (2024_EKEA.104), and a grant from the Federal Ministry of Education and Research (Bundesministerium für Bildung und Forschung, BMBF) as part of the German Center for Child and Adolescent Health (DZKJ) under the funding code 01GL2405B.

## Declaration of competing interest

The authors declare that they have no known competing financial interests or personal relationships that could have appeared to influence the work reported in this paper.

## Data Availability

Due to ethical considerations, the data that support the findings of this study are only available from the corresponding author, upon reasonable request.
