# Supplementary material for: Altered neural oscillatory dynamics underlie reduced anticipatory schema use during event segmentation in adolescents with High-Functioning Autism Spectrum disorder
Source: Neuroimage Clin. 2026 Apr 26;50:103998. doi: 10.1016/j.nicl.2026.103998 (PMC13141802; doi:10.1016/j.nicl.2026.103998)
Supplement: Supplementary Data 1 [file mmc1.docx]

**SUPPLEMENTAL MATERIAL**

**Altered Neural Oscillatory Dynamics Underlie Reduced Anticipatory Schema Use During Event Segmentation in Adolescents with High-Functioning Autism Spectrum Disorder**

Ronja Limburg, Michel Benjamin Kopp, Xianzhen Zhou, Foroogh Ghorbani, Veit Roessner, Bernhard Hommel, Christian Beste, Astrid Prochnow

**Supplemental Participant Information**

*Supplemental Table 1*. Overview of comorbid psychiatric conditions in the ASD sample.

| **ICD-10 code** | **Condition** | **Prevalence (n)** |
| --- | --- | --- |
| F43.2 | disorder of adjustment | 1 |
| F74 | dissociated intelligence | 1 |
| F80.0 | phonological disorder | 1 |
| F80.1 | expressive language disorder | 2 |
| F80.2 | mixed receptive-expressive language disorder | 1 |
| F80.20 | disorder of auditory processing and perception | 1 |
| F81.0 | dyslexia | 2 |
| F81.2 | dyscalculia | 1 |
| F82 | specific developmental disorder of motor function | 8 |
| F82.0 | developmental disorder of gross motor skills | 2 |
| F82.1 | developmental disorders of fine and graphomotor skills | 1 |
| F90.0 | attention-deficit hyperactivity disorder | 6 |
| F90.2 | hyperkinetic disorder with conduct disorder | 1 |
| F93.8 | other childhood emotional disorders | 1 |
| F93.9 | childhood emotional disorders, unspecified | 1 |
| F98.8 | attention-deficit without hyperactivity | 1 |
| F98.80 | other specified behavioral and emotional disorders with onset in childhood | 1 |

Diagnoses were ascertained by clinicians. N = 11 ASD participants presented with no comorbidities.

**Supplemental Analysis 1: Group Differences in EEG Data Quality**

To check whether participant groups differed with regard to EEG data quality, we compared the number of channels that were interpolated in the course of EEG preprocessing. With a mean number of 11.1 (± 3.6) interpolated channels in the NT group and a mean number of 9.6 (± 3.4) interpolated channels in the ASD group, group differences were not significant (*t*(58) = 1.70, *p* = .094). Yet, the number of reconstructed time points due to noisy data was significantly lower in the NT than in the ASD group (*t*(58) = -2.58, *p* = .013). The mean length of all reconstructed samples accumulated to 41.7 (± 77.1) sec in the ASD group and 5.4 (± 5.3) sec in the NT group. However, as the removal of reconstructed samples was performed before segmentation, it is unclear whether the affected samples correspond to time points during the task or during breaks. For this reason, we additionally compared the number of valid Boundary trials retained in the EEG data between groups. Mean number of Boundary trials with retained EEG data was 58.2 (± 42.8) in the NT group and 64.4 (± 60.0) in the ASD group. Mirroring the observation on the behavioral level, the number of valid Boundary trials in the EEG data after removal of noisy segments did not differ between groups (*t*(58) = -0.46, *p* = .645). Consequently, group differences in the EEG data quality are unlikely to have biased our results.

**Supplemental Analysis 2: Sensor Level Analysis of Oscillatory Activity**

To compare sensor level activity profiles of ASD and NT participants across EEG frequency bands, we first performed a wavelet-based time-frequency analysis (Morlet wavelet with length of 3 SD of the implicit Gaussian kernel, frequency range: 3-30 Hz, number of cycles increasing linearly from 3 to 12), using the FieldTrip toolbox (Oostenveld et al., 2011), to decompose the EEG signal into theta (4-7 Hz), alpha (8-12 Hz) and beta (15-30 Hz) band activity. Although we were interested in effects occurring in the -/+1 sec interval surrounding responses or virtual markers, we applied the time-frequency analysis on a time window encompassing -/+2 sec around respective markers to prevent edge effects. Data in the first and last 1 sec of the resulting power spectrum were discarded prior to further analyses. The subsequent analyses were performed separately for the pre and post response time window and involved a cluster-based permutation test (CBPT; number of permutations: 1 000, *p*-value corrected at cluster level) that contrasted average power estimates during BI and NBI within each group. By application of this CBPT, we identified in which frequency bands activity was linked to the identification of event boundaries. In addition, we subtracted activity in NBI from activity in BI for each group and conducted a between-groups CBPT to test whether the activity modulation between conditions was significantly different between ASD and NT adolescents.

*Results*

**Pre-boundary.** In the pre-boundary period, activity differences between BI and NBI in theta band activity (TBA) were limited to the NT group (among two positive clusters observed, one was significant, see Suppl. Tab. 2 for cluster statistics), while no TBA cluster was observed for the ASD group, Suppl. Fig. 1.

*Supplemental Table 2*. Cluster statistics for all significant clusters observed in the CBPT contrasting oscillatory activity between BI and NBI on sensor level.

|  |  | **NT** | | **ASD** | |
| --- | --- | --- | --- | --- | --- |
| **Time window** | **Frequency Band** | **T_sum_** | ***p*** | **T_sum_** | ***p*** |
| Pre-response | TBA | 14.75 | .034 | / | / |
| Pre-response | ABA | -156.38 | .002 | -89.95 | .002 |
| Pre-response | BBA | -138.02 | .002 | -119.67 | .002 |
| Post-response | TBA | -17.97 | .032 | -24.43 | .012 |
| Post-response | ABA | -249.82 | .002 | −210.89 | .002 |
| Post-response | BBA | -94.06 | .002 | -118.43 | .002 |

TBA = theta band activity, ABA = alpha band activity, BBA = beta band activity.

Yet, the ASD and NT group alike exhibited significant condition-dependent activity modulations in alpha band activity (ABA) and beta band activity (BBA), as indicated by our finding of a negative cluster in ABA and a negative cluster in BBA. However, when comparing these condition-specific activity differences between groups, no clusters were found in any of the investigated frequency bands.

**Post-boundary.** In the post-boundary time window, both groups exhibited significant activity differences between BI and NBI in all investigated frequency bands. For TBA, one non-significant positive and one significant negative cluster were present in the NT group, while TBA modulations in the ASD group were limited to a single negative cluster. Furthermore, in both groups one negative cluster in ABA and one negative cluster in BBA were apparent. The comparison of these condition specific-activity differences, again, yielded no group differences.


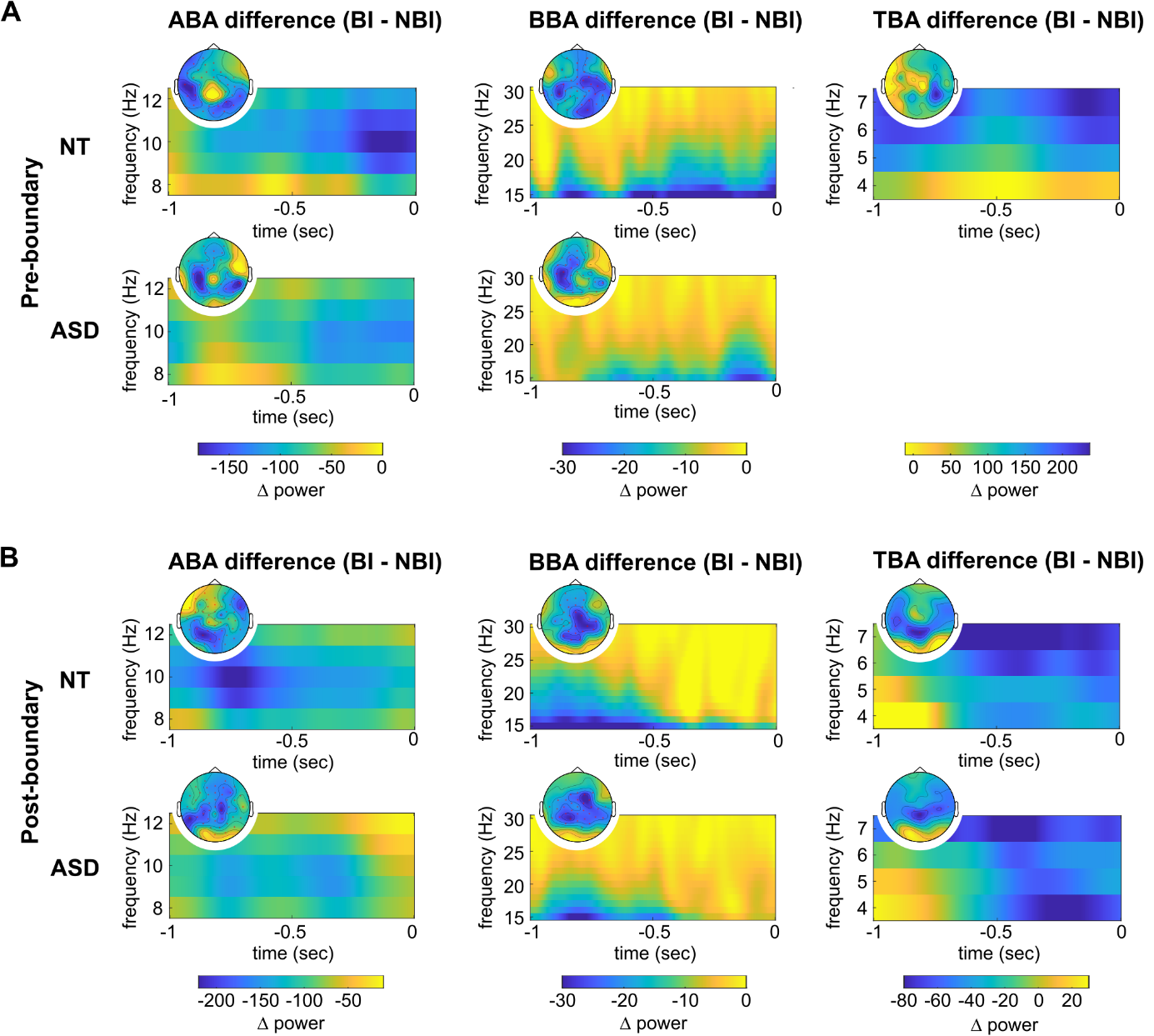


*Supplemental Figure 1 – Sensor level neurophysiological results for the pre- and post-boundary period*. Time frequency analyses results (time-frequency plot) and cluster-based permutation test results (topographic plot) for frequencies in which significant activity differences between Boundary (BI) and No-Boundary (NBI) intervals was observed (ABA: first column; BBA: second column, TBA: third column). Time-frequency plots and topographic plots display the within-group mean power difference (BI minus NBI) averaged across the significant electrodes identified through cluster-based permutation testing. Location of the significant electrodes is highlighted in red on the topographic plots. Panel A illustrates results for the pre-boundary period, while panel B shows results for the post-boundary period. In each panel, results are displayed separately for the NT group (first row) and the ASD group (second row).

**Supplemental Analysis 3: Movie Liking**

After completing the segmentation task, participants were asked to rate if they liked the RedBalloon movie. Answers were given on a Likert scale ranging from 1 (I agree) to 5 (I do not agree). The rating from N = 1 ASD participant was not available. Ratings were compared between groups by means of a Wilcoxon ranksum test (function ‘ranksum’ in MATLAB R2022a) to account for the possibility that disliking the movie might reduce task engagement.

The Wilcoxon ranksum test applied to participants ratings of how much they liked the RedBalloon movie revealed no group differences, (*Z* = 1.03*, p* = .305). In the NT group, the mean likeability rating was at 2.93 ($\pm$ 1.05) and in the ASD group at 2.59 ($\pm$ 1.30).

**Supplemental Analysis 4: Segmentation Agreement**

To rule out the possibility that ASD participants might have responded at random, we calculated an agreement score for the timing of event boundaries set by ASD compared to NT participants. To accomplish this, we first generated a segmentation norm based on the responses of NT participants. Responses of all NT participants were available in the format of a time series consisting of 2 sec bins (number of bins: 668). To each bin either a 0 (no boundary indicated) or a 1 (boundary indicated) was assigned. To characterize the segmentation behavior in the NT group we calculated the minimum, mean, maximum and standard deviation of the number of indicated event boundaries across all NT participants. This information was used to generate 1000 simulated datasets with the same characteristics as the NT data (i.e. same mean number of boundaries, same standard deviation of the boundary number and boundaries per dataset lying within the minimum and maximum values from the NT sample). Within the simulated datasets, the location of boundaries was randomly distributed over the time course of the movie, to reflect unsystematic segmentation behavior. For each time bin, we then calculated the percentage agreement between our 1000 simulated datasets by counting how many simulated datasets included an event boundary within the same time bin and dividing this sum by the number of datasets (i.e. 1000). The agreement values for all time bins were then sorted by size in ascending order, to determine the highest agreement value occurring in less than 5% of all cases. This value was defined to be a minimum agreement threshold to assume the existence of normative event boundaries. After calculating the agreement value for each time bin across all NT participants, we applied our threshold to find time bins surpassing our minimum agreement criterion and marked these bins as normative boundary locations.

In the second step, we used these normative boundary locations to investigate how well the responses of ASD participants aligned with the NT norm. We did this, by calculating the point biserial correlation (Nagel, 2006) between each of our ASD participants’ response vector and the NT norm. Hence, we obtained one correlation coefficient per ASD participant, as displayed in Supplemental Table 3.

*Supplemental Table 3.* Results from the agreement analysis evaluating how well the timing of indicated event boundaries across the movie aligned between the NT norm and each ASD participant.

| **ASD participant number** | **Correlation coefficient** | **Correlation *p*-value** |
| --- | --- | --- |
| ASD_01 | 0.17 | < .001 |
| ASD_02 | 0.16 | < .001 |
| ASD_03 | 0.18 | < .001 |
| ASD_04 | 0.08 | .038 |
| ASD_05 | 0.20 | < .001 |
| ASD_06 | 0.00 | .935 |
| ASD_07 | 0.06 | .132 |
| ASD_08 | 0.24 | < .001 |
| ASD_09 | 0.13 | .001 |
| ASD_10 | 0.19 | < .001 |
| ASD_11 | 0.23 | < .001 |
| ASD_12 | 0.29 | < .001 |
| ASD_13 | 0.11 | .006 |
| ASD_14 | 0.05 | .213 |
| ASD_15 | 0.14 | < .001 |
| ASD_16 | 0.14 | < .001 |
| ASD_17 | 0.10 | .012 |
| ASD_18 | 0.09 | .014 |
| ASD_19 | 0.21 | < .001 |
| ASD_20 | 0.12 | .003 |
| ASD_21 | 0.17 | < .001 |
| ASD_22 | 0.28 | < .001 |
| ASD_23 | 0.12 | .001 |
| ASD_24 | 0.15 | < .001 |
| ASD_25 | 0.15 | < .001 |
| ASD_26 | 0.18 | < .001 |
| ASD_27 | 0.06 | .143 |
| ASD_28 | 0.17 | < .001 |
| ASD_29 | 0.10 | .008 |
| ASD_30 | 0.05 | .222 |

**Supplemental Result Tables**

*Supplemental Table 4*. Results from the regression analysis predicting segmentation from the type of change and group membership (0 = NT, 1 = ASD).

| **Predictor** | **Estimate** | **95% CrI** | **OR** | **OR 95% CI** |
| --- | --- | --- | --- | --- |
| Intercept | -3.21 | -3.44 to -2.99 | 0.04 | 0.03 to 0.05 |
| Character | 0.60 | 0.46 to 0.73 | 1.82 | 1.59 to 2.07 |
| CharacterCharacter | 0.28 | 0.12 to 0.44 | 1.32 | 1.12 to 1.55 |
| CharacterObject | 0.53 | 0.36 to 0.70 | 1.71 | 1.43 to 2.01 |
| Temporal | 0.51 | 0.26 to 0.76 | 1.67 | 1.30 to 2.15 |
| Large Space | 0.32 | 0.10 to 0.54 | 1.38 | 1.11 to 1.72 |
| Small Space | 0.44 | 0.30 to 0.57 | 1.55 | 1.35 to 1.76 |
| Cause | 0.39 | 0.22 to 0.55 | 1.48 | 1.25 to 1.73 |
| Goal | -0.20 | -0.38 to -0.03 | 0.82 | 0.69 to 0.97 |
| Scene | -0.24 | -0.43 to -0.05 | 0.79 | 0.65 to 0.95 |
| Character*ASD | -0.07 | -0.26 to 0.11 | 0.93 | 0.77 to 1.11 |
| CharacterCharacter*ASD | 0.03 | -0.20 to 0.26 | 1.03 | 0.82 to 1.29 |
| CharacterObject*ASD | -0.07 | -0.31 to 0.17 | 0.93 | 0.73 to 1.18 |
| Temporal*ASD | -0.03 | -0.40 to 0.35 | 0.97 | 0.67 to 1.42 |
| Large Space*ASD  Small Space*ASD  Cause*ASD  Goal*ASD  Scene*ASD | -0.24  -0.22  -0.20  0.03  0.02 | -0.57 to 0.07  -0.40 to -0.04  -0.44 to 0.04  -0.21 to 0.27  -0.25 to 0.28 | 0.79  0.80  0.82  1.03  1.01 | 0.57 to 1.07  0.67 to 0.97  0.65 to 1.04  0.81 to 1.31  0.78 to 1.32 |

CrI = 95% credibility interval for the model estimate; OR = Odds Ratio; CI = 95% Odds Ratio confidence interval.

*Supplemental Table 5.* Convergence Diagnostics for the logistic regression model estimating segmentation likelihood as a function of the number of changes and group membership (0 = NT, 1 = ASD).

| **Predictor** | **R-hat** | **Bulk ESS** | **Tail ESS** |
| --- | --- | --- | --- |
| Intercept | 1.01 | 464 | 838 |
| Number of Changes | 1.00 | 4667 | 4392 |
| ASD | 1.00 | 509 | 789 |
| Number of Changes*ASD | 1.00 | 4447 | 4153 |

ESS = effective sample size, Model based on four Markov chain Monte Carlo (MCMC) chains with 3000 iterations each, 1500 warmup draws and 6000 post-warmup draws with default prior settings (i.e. flat priors for fixed effects and weakly informative student-t priors for the intercept). R-hat indicates if all Markov chain Monte Carlo (MCMC) chains can be assumed to be drawn from the same underlying distribution by contrasting the between and within-chain estimates for model parameters. In the ideal case, R-hat = 1. For the bulk and tail ESS it is recommended to reach an ESS of least 100 per chain (Vehtari et al., 2021).

*Supplemental Table 6.* Convergence Diagnostics for the logistic regression model estimating segmentation likelihood as a function of the types of changes and group membership (0 = NT, 1 = ASD).

| **Predictor** | **R-hat** | **Bulk ESS** | **Tail ESS** |
| --- | --- | --- | --- |
| Intercept | 1.02 | 714 | 1575 |
| Character | 1.00 | 7295 | 7558 |
| CharacterCharacter | 1.00 | 7005 | 7097 |
| CharacterObject | 1.00 | 8249 | 7414 |
| Temporal | 1.00 | 8116 | 7550 |
| Large Space | 1.00 | 5354 | 7123 |
| Small Space | 1.00 | 7673 | 7801 |
| Cause | 1.00 | 8046 | 7679 |
| Goal | 1.00 | 7363 | 7608 |
| Scene | 1.00 | 5443 | 6972 |
| Character*ASD | 1.00 | 7041 | 7200 |
| CharacterCharacter*ASD | 1.00 | 7019 | 7614 |
| CharacterObject*ASD | 1.00 | 8420 | 7734 |
| Temporal*ASD | 1.00 | 7826 | 7614 |
| Large Space*ASD  Small Space*ASD  Cause*ASD  Goal*ASD  Scene*ASD | 1.00  1.00  1.00  1.00  1.00 | 5330  7642  8279  7104  5282 | 7087  7312  7874  8059  7326 |

ESS = effective sample size, Model based on four Markov chain Monte Carlo (MCMC) chains with 5000 iterations each, 2500 warmup draws and 10000 post-warmup draws with default prior settings (i.e. flat priors for fixed effects and weakly informative student-t priors for the intercept).

**Supplemental Analysis 5: Posterior Predictive Checks for the Bayesian Regression Models**

To evaluate the performance of our Bayesian logistic regression models, we performed posterior predictive checks using the pp_check function (Kleiber & Zeileis, 2016) provided in R. This function offers several options to evaluate whether data simulated based on the Bayesian model is similar to the observed data. Posterior predictive checks for the Bayesian model evaluating the influence of the number of changes and group membership on segmentation likelihood are provided in Supplemental Figure 2 and for the model testing the predictive value of the types of situational changes and group membership on segmentation probability in Supplemental Figure 3. In both figures, each panel illustrates the binned residuals computed from the difference between one simulated dataset and the observed data. The residuals are binned according to the predicted probabilities (x-axis) which indicate the chance of an event boundary being set (i.e. the model outcome equaling 1) given a certain specification of the predictor values. Positive errors indicate underprediction while negative errors indicate overprediction by the models. As shown in the figures, no systematic pattern of over- or underprediction is evident at any predicted probability, suggesting good calibration of the models. Slight increases in the error magnitudes in the higher probability ranges are to be expected as they are based on fewer observations, and the errors are still mostly located within the uncertainty bounds (light blue shaded areas). Based on the observation of similar residual error patterns across the 10 posterior draws we can also assume that the Bayesian models are rather stable. Overall, the posterior predictive checks, thus, suggest the absence of major model misspecifications or biases.


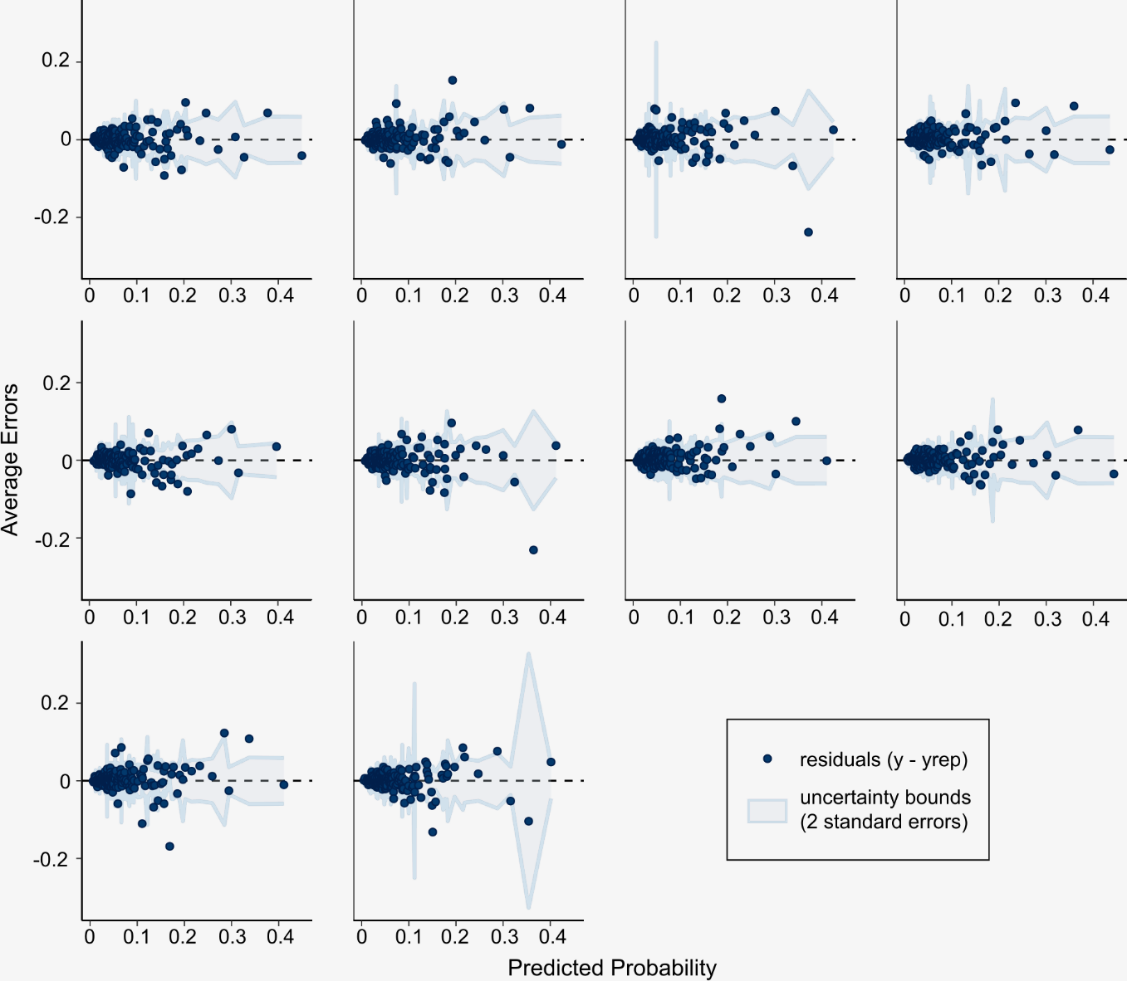


*Supplemental Figure 2 - Posterior predictive check for the logistic regression model estimating segmentation likelihood as a function of the number of changes and group membership.* Each panel represents the residuals resulting from the comparison between the observed data (y) and predicted data (yrep). The residuals are binned along the x-axis according to the predicted probability.


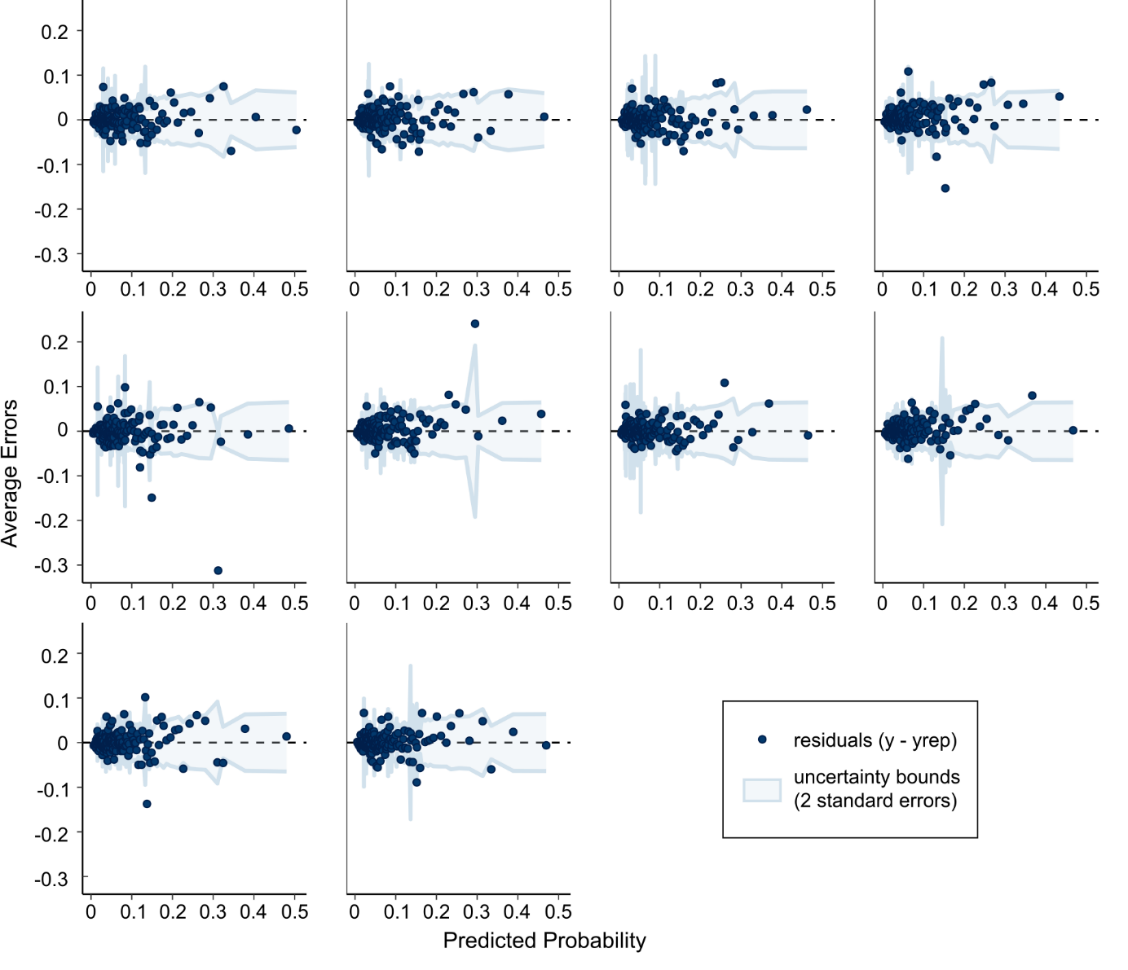


*Supplemental Figure 3 - Posterior predictive check for the logistic regression model estimating segmentation likelihood as a function of the types of situational changes and group membership.* Each panel represents the residuals resulting from the comparison between the observed data (y) and one posterior draw (yrep). The residuals are binned along the x-axis according to the predicted probability.

**Supplemental Analysis 6: Sensitivity Analysis for Different DBSCAN Thresholds**

To assess the robustness of the neuroanatomical origins of greatest activity modulations as identified at a DBSCAN threshold of 1%, we additionally applied the DBSCAN with the threshold set to 0.5% and 2%. A comparative overview of the regions implicated at each thresholding level is provided in Supplemental Tables 7 and 8 and Supplemental Figure 4 for the pre-response time window, as well as in Supplemental Tables 9 and 10 and Supplemental Figure 5 for the post-response time window.

*Supplemental Table 7*. Anatomical locations of largest pre-response activity modulations at 0.5%, 1% and 2% DBSCAN thresholding in the neurotypical (NT) group.

|  | **Threshold 0.5%** | **Threshold 1%** | **Threshold 2%** |
| --- | --- | --- | --- |
| **ABA (negative clusters)** | Cluster 1:  inferior parietal (L)  precentral (L)  postcentral (L) | Cluster 1:  fusiform gyrus (R)  inferior occipital (R)  inferior temporal (R)  Cluster 2:  inferior parietal (L)  supramarginal (L)  precentral (L)  postcentral (L) | Cluster 1:  fusiform gyrus (R)  inferior occipital (R)  inferior temporal (R)  Cluster 2:  superior occipital (R)  calcarine (R)  cuneus (R)  middle occipital (R)  Cluster 3:  inferior parietal (L)  supramarginal (L)  precentral (L)  postcentral (L)  Cluster 4:  inferior parietal (R)  superior parietal (R)  postcentral (R) |
| **BBA (negative clusters)** | Cluster 1:  inferior parietal (L)  precentral (L)  postcentral (L) | Cluster 1:  inferior parietal (L)  supramarginal (L)  precentral (L)  postcentral (L) | Cluster 1:  inferior parietal (L)  supramarginal (L)  precentral (L)  postcentral (L)  middle frontal (L)  superior frontal (L) |

In the NT group no theta clusters were revealed in the leave-one-out cluster-based permutation test; TBA = theta band activity; ABA = alpha band activity; BBA = beta band activity; (L) = left-lateralized; (R) = right-lateralized; (B) = bilateral.

*Supplemental Table 8.* Anatomical locations of largest pre-response activity modulations at 0.5%, 1% and 2% DBSCAN thresholding in the ASD group.

|  | **Threshold 0.5%** | **Threshold 1%** | **Threshold 2%** |
| --- | --- | --- | --- |
| **TBA (positive clusters)** | Cluster 1:  inferior temporal (L)  Cluster 2 and 3:  medial superior frontal (L)  superior frontal (L) | Cluster 1:  inferior temporal (L)  fusiform gyrus (L)  Cluster 2:  medial superior frontal (L)  superior frontal (L) | Cluster 1:  inferior temporal (L)  fusiform gyrus (L)  Cluster 2:  superior temporal (L)  Heschl’s gyrus (L)  Rolandic operculum (L)  precentral (L)  postcentral (L)  Cluster 3:  superior temporal (R)  Cluster 4:  medial superior frontal (B)  superior frontal (L)  anterior cingulum (L)  middle frontal (L) |
| **ABA (negative clusters)** | Cluster 1:  inferior parietal (L)  supramarginal (L)  precentral (L)  postcentral (L) | Cluster 1:  inferior parietal (L)  supramarginal (L)  precentral (L)  postcentral (L) | Cluster 1  inferior parietal (L)  supramarginal (L)  precentral (L)  postcentral (L)  middle frontal (L)  inferior frontal gyrus pars triangularis (L) |
| **BBA (negative clusters)** | Cluster 1:  middle frontal (L)  superior frontal (L)  precentral (L)  postcentral (L) | Cluster 1:  middle frontal (L)  superior frontal (L)  precentral (L)  postcentral (L)  supramarginal (L) | Cluster 1:  middle frontal (L)  superior frontal (L)  precentral (L)  postcentral (L)  supramarginal (L)  supplemental motor area (L) |

TBA = theta band activity; ABA = alpha band activity; BBA = beta band activity; (L) = left-lateralized; (R) = right-lateralized; (B) = bilateral.


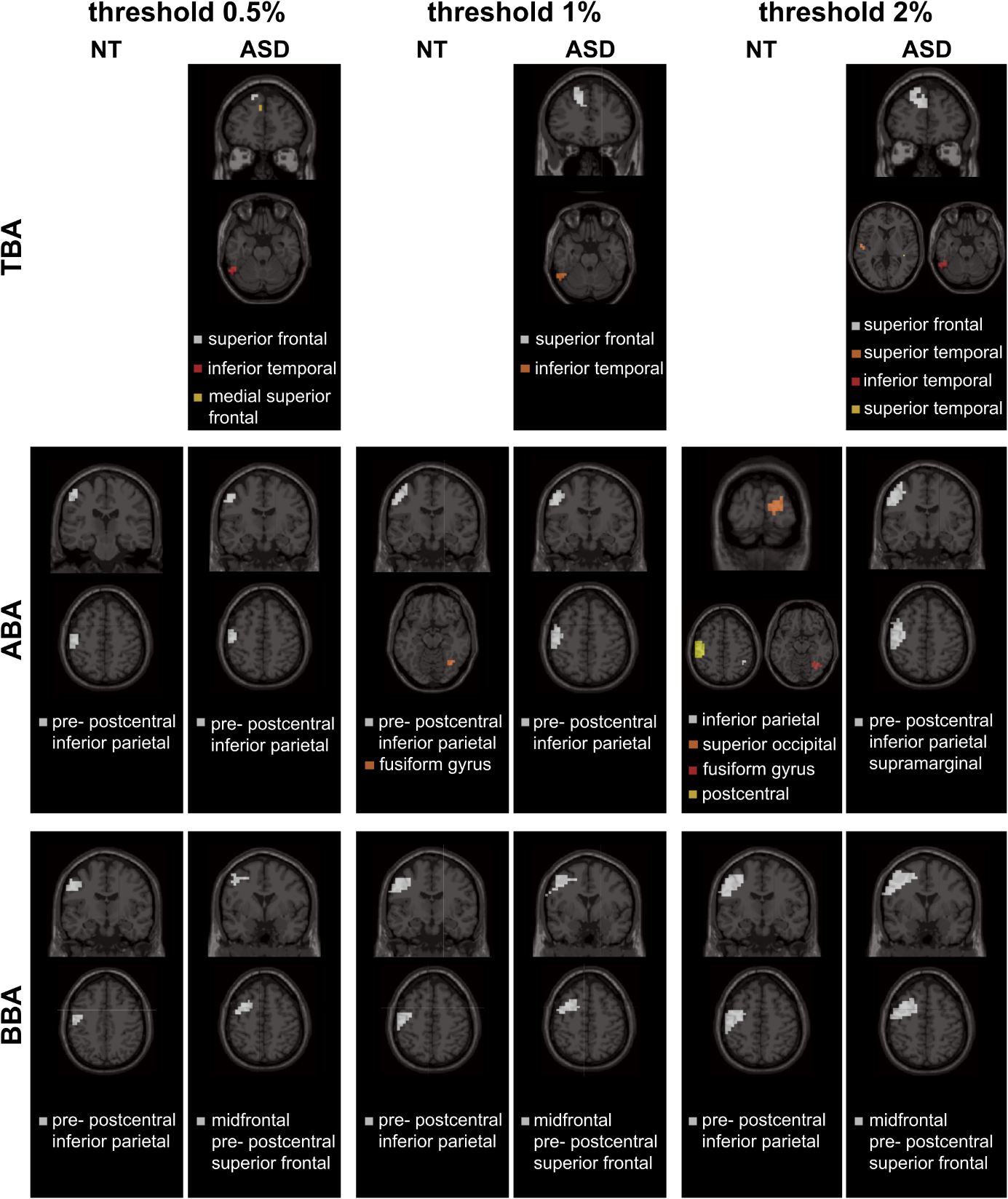


*Supplemental Figure 4 - Neuroanatomical regions exhibiting the top 0.5%, 1% and 2% activity modulations in the pre-response time window.* In each column, voxels exhibiting the greatest pre-response activity difference (BI minus NBI) are accentuated. Each column visualizes the regions implicated at different DBSCAN thresholding levels (left column: voxels exhibiting the top 0.5% activity difference, middle: voxels exhibiting the top 1% activity difference; right: voxels exhibiting the top 2% activity difference). Different colors are used to mark distinct clusters. Depiction of TBA modulations in NT participants is omitted as significant clusters for the BI – NBI contrast were absent. The full list of regions constituting the shown clusters is provided in Supplemental Tables 4 and 5.

*Supplemental Table 9.* Anatomical locations of largest pre-response activity modulations at 0.5%, 1% and 2% DBSCAN thresholding in the neurotypical (NT) group.

|  | **Threshold 0.5%** | **Threshold 1%** | **Threshold 2%** |
| --- | --- | --- | --- |
| **TBA (negative clusters)** | Cluster 1:  superior frontal (R)  superior orbitofrontal (R)  medial superior frontal (R)  Cluster 2:  superior frontal (R)  precentral (R)  supplemental motor area (R) | Cluster 1:  superior frontal (R)  medial superior frontal (R)  superior orbitofrontal (R)  medial orbitofrontal (R)  Cluster 2:  Rolandic operculum (R)  frontal inferior operculum (R)  superior temporal pole (R)  Cluster 3:  superior frontal (R)  precentral (R)  postcentral (R)  supplemental motor area (R) | Cluster 1:  superior frontal (R)  medial superior frontal (R)  superior orbitofrontal (R)  medial orbitofrontal (R)  middle frontal (R)  Cluster 2:  Rolandic operculum (R)  frontal inferior operculum (R)  superior temporal pole (R)  precentral (R)  Cluster 3:  superior frontal (R)  precentral (R)  postcentral (R)  supplemental motor area (R) |
| **ABA (negative clusters)** | Cluster 1:  precentral (L)  postcentral (L)  inferior parietal (L) | Cluster 1:  precentral (L)  postcentral (L)  inferior parietal (L)  supramarginal (L)  superior frontal (L) | Cluster 1:  precentral (L)  postcentral (L)  inferior parietal (L)  supramarginal (L)  superior frontal (L)  Cluster 2:  precentral (R)  middle frontal (R)  superior frontal (R) |
| **BBA (negative clusters)** | Cluster 1:  precentral (L)  Cluster 2:  superior frontal (R) | Cluster 1:  superior frontal (R)  precentral (R)  postcentral (R)  Cluster 2:  precentral (L)  Cluster 3:  postcentral (R) | Cluster 1:  superior frontal (R)  middle frontal (R)  precentral (R)  postcentral (R)  superior parietal (R)  inferior parietal (R)  Cluster 2:  superior frontal (L)  precentral (L)  postcentral (L)  Cluster 3:  superior frontal (L)  paracentral lobule (L)  supplemental motor area (L) |

TBA = theta band activity; ABA = alpha band activity; BBA = beta band activity; (L) = left-lateralized; (R) = right-lateralized.

*Supplemental Table 10.* Anatomical locations of largest pre-response activity modulations at 0.5%, 1% and 2% DBSCAN thresholding in the ASD group.

|  | **Threshold 0.5%** | **Threshold 1%** | **Threshold 2%** |
| --- | --- | --- | --- |
| **ABA (negative clusters)** | Cluster 1:  precentral (L)  postcentral (L)  inferior parietal (L)  supramarginal (L)  superior frontal (L) | Cluster 1:  precentral (L)  postcentral (L)  inferior parietal (L)  supramarginal (L)  superior frontal (L)  middle frontal (L) | Cluster 1:  precentral (L)  postcentral (L)  inferior parietal (L)  supramarginal (L)  superior frontal (L)  middle frontal (L)  Cluster 2:  middle frontal (R)  superior frontal (R)  precentral (R) |

In ASD no TBA or BBA clusters were observed in the leave-one-out cluster-based permutation test; TBA = theta band activity; ABA = alpha band activity; BBA = beta band activity; (L) = left-lateralized; (R) = right-lateralized.


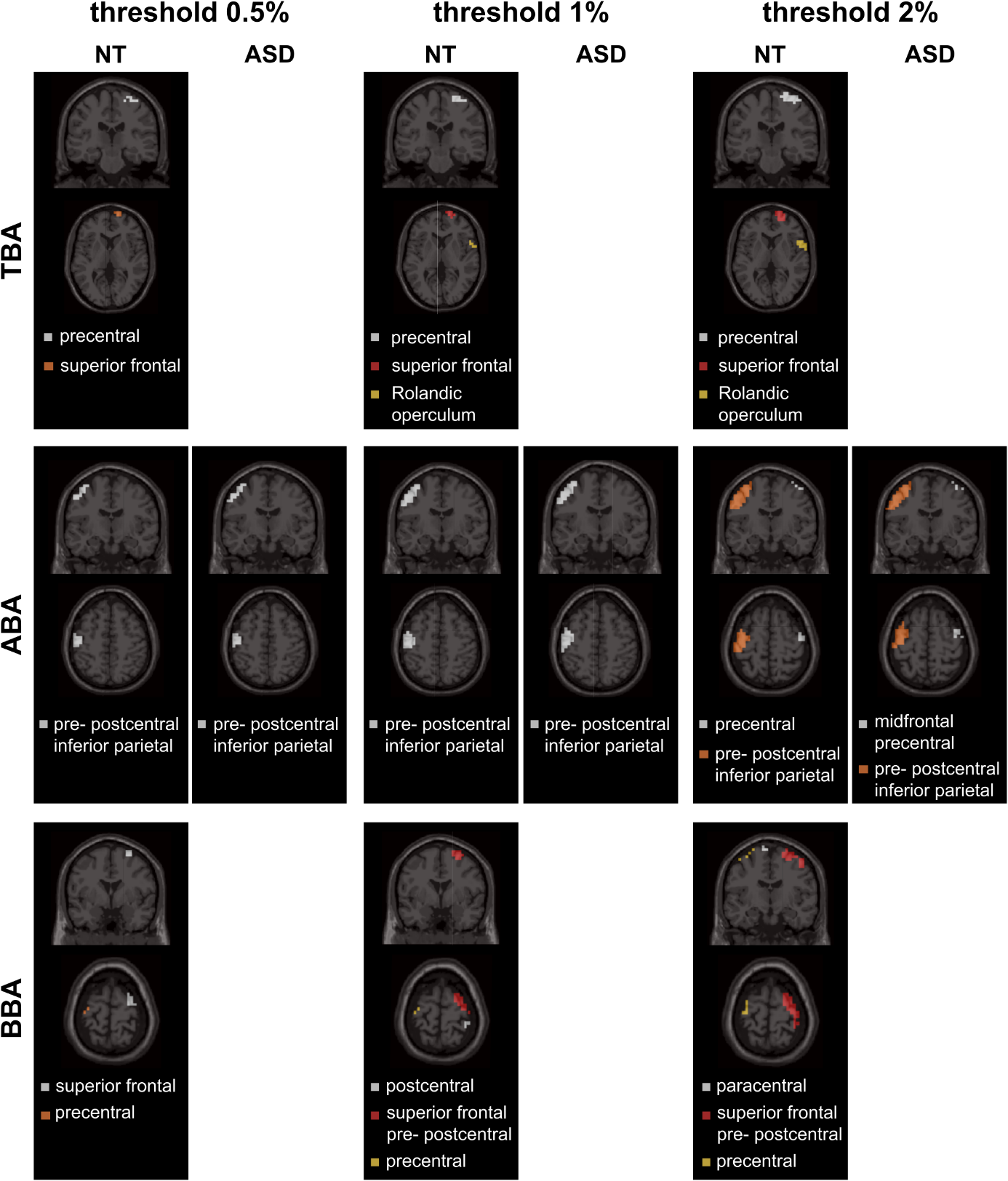


*Supplemental Figure 5 - Neuroanatomical regions exhibiting the top 0.5%, 1% and 2% activity modulations in the post-response time window.* In each column, voxels exhibiting the greatest post-response activity difference (BI minus NBI) are accentuated. Each column visualizes the regions implicated at different DBSCAN thresholding levels (left column: voxels exhibiting the top 0.5% activity difference, middle: voxels exhibiting the top 1% activity difference; right: voxels exhibiting the top 2% activity difference). Different colors are used to mark distinct clusters. Depiction of ABA and BBA modulations in NT participants is omitted as significant clusters for the BI – NBI contrast were absent. The full list of regions constituting the shown clusters is provided in Supplemental Tables 6 and 7.

**References**

Kleiber, C., & Zeileis, A. (2016). Visualizing Count Data Regressions Using Rootograms. *The American Statistician*, *70*(3), 296–303. https://doi.org/10.1080/00031305.2016.1173590

Nagel, F. (2006). *Point biserial correlation* [Computer software]. https://www.mathworks.com/matlabcentral/fileexchange/11222-point-biserial-correlation

Oostenveld, R., Fries, P., Maris, E., & Schoffelen, J.-M. (2011). FieldTrip: Open Source Software for Advanced Analysis of MEG, EEG, and Invasive Electrophysiological Data. *Computational Intelligence and Neuroscience*, *2011*(1), 156869. https://doi.org/10.1155/2011/156869
